# Benefits of surgery in the multimodality treatment of stage IIB-IIIC small cell lung cancer

**DOI:** 10.7150/jca.31202

**Published:** 2019-08-29

**Authors:** Yong Yang, Guangda Yuan, Cheng Zhan, Yiwei Huang, Mengnan Zhao, Xiaodong Yang, Shuai Wang, Zongwu Lin, Shiying Zheng, Tao Lu, Weigang Guo, Qun Wang

**Affiliations:** 1 Department of Cardio-Thoracic Surgery, First Affiliated Hospital of Soochow University, Suzhou, Jiangsu Province, China; 2 Department of Thoracic Surgery, Suzhou Hospital affiliated to Nanjing Medical University, Suzhou, Jiangsu Province, China; 3 Department of Thoracic Surgery, Zhongshan Hospital, Fudan University, Shanghai, China

**Keywords:** small cell lung cancer, surgery, non-surgery, propensity score matching, survival

## Abstract

Surgery combined with chemotherapy/radiotherapy is recommended for early stage small cell lung cancer (SCLC); however, the role of surgery in the multimodality treatment of advanced disease remains controversial. The clinical data of patients between 2000 and 2015 were obtained from the Surveillance, Epidemiology, and End Results database. The surgery group included 998 patients with stage IIB-IIIC. A matched non-surgery group (n = 2994) was generated by propensity score matching. The Kaplan-Meier method and log-rank tests were used for survival analyses. Univariate and multivariate analyses were used to identify significant prognostic factors. After matching, there were no significant differences between the two groups in race, age, sex, T classification, N classification, TNM stage, marital status, primary sites, and origin record NAACCR Hispanic Identification Algorithm (NHIA). The surgery group showed better overall survival and cancer-specific survival than the non-surgery group. Univariate and multivariate analyses showed that therapy methods, age, sex, T classification, and N classification were independent prognostic predictors for stage IIB-IIIC SCLC (all *P* < 0.05). Stratified analyses showed that survival outcomes favored surgery in any age groups, men and women, any T classification except T3, and N0-2 but not N3 compared with non-surgical treatment. The survival differences favored surgery in stage IIB and IIIA SCLC, although they were not significant in stage IIB and IIIC SCLC. Therefore, surgery was associated with improved survival in stage IIB and IIIA SCLC, but not in stage IIIB and IIIC SCLC.

## Introduction

Small cell lung cancer (SCLC) accounts for approximately 15% of new lung cancer cases annually. It is a virulent, rapidly growing, early metastasizing, and highly invasive cancer with a 5-year survival rate of < 7% 1, 2. Most of patients with SCLC already have regional or distant spread at diagnosis 3. Despite recent advances in the treatment of SCLC, patient survival only shows a slight improvement 4.

Currently, chemotherapy and radiotherapy are the standard treatments for patients with SCLC, and they have provided patients a chance to prolong their survival 5. Surgical treatment is only considered to be limited as an option for early stage SCLC according to recent consensus. The NCCN guidelines version 2 (2018) suggest that patients with SCLC at a stage higher than T1-2 with N0 do not benefit from surgery 6. However, reports show that surgery can significantly improve survival outcomes in SCLC patients and should be considered in the management of SCLC at stages higher than T1-2 with N0 7.

The role of surgery in the multimodality treatment of advanced SCLC remains controversial. Here, we investigated whether patients with stage IIB-IIIC SCLC could benefit from surgical resection as part of the multimodality treatment based on chemotherapy/radiotherapy.

## Material and Methods

### Data acquisition

The data of patients with small lung cancer were obtained from the Surveillance, Epidemiology, and End Results (SEER) database (Incidence—SEER 18 Regs Custom Data with additional treatment fields, Nov 2017 Sub, 1973 - 2015 varying) via SEER∗Stat, version 8.3.5 (http://seer. cancer.gov/seerstat/).

**Figure 1:**
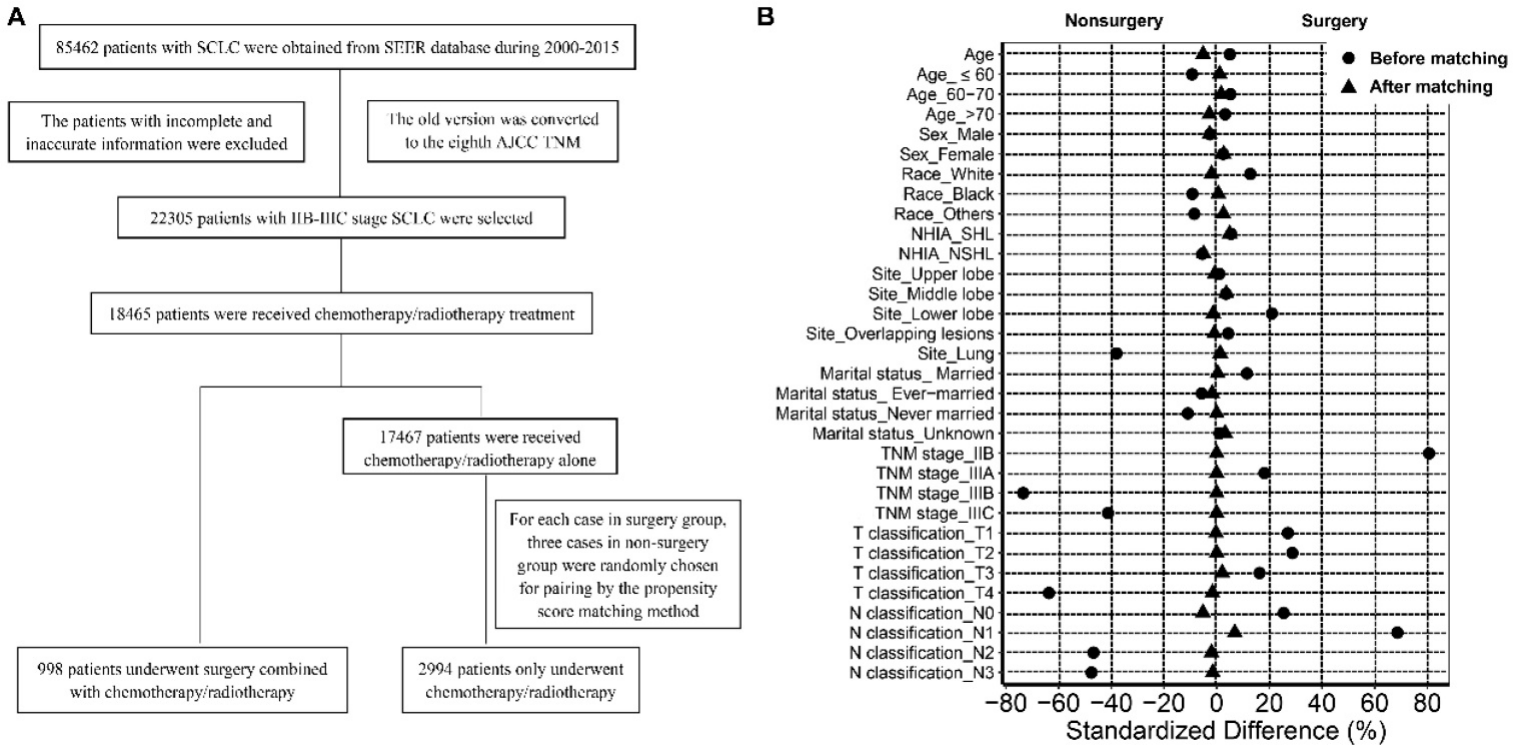
Flow chart of the selection of cases and controls (A); Standardized differences of baseline variables before and after propensity score matching (B).

The data of patients with SCLC are shown in Figure 1A. Briefly, patients with SCLC (ICD-O-3 8041/3, 8043/3, 8044/3, 8045/3) were recruited between 2000 and 2015. The labeled primary sites were limited to C34.1-upper lobe, lung, C34.2-middle lobe, lung, C34.3-lower lobe, lung, and C34.8-Overlapping lesion of lung, C34.9-lung, NOS. The old version of tumor TNM stage was converted to the new AJCC TNM stage manually. The inclusion criteria were as follows: (1) Patients with stage IIB-IIIC disease; and (2) patients treated with chemotherapy or radiotherapy or both. The exclusion criteria were as follows: (1) Patients diagnosed with stage I, IIA, and IV SCLC; (2) patients who did not receive chemotherapy/radiotherapy or surgery; (3) patients who underwent surgical treatment alone. After selection, all patients enrolled in the study were divided into a surgery group and a non-surgery (chemotherapy/radiotherapy) group according to the mode of therapy.

The data of patient and tumor characteristics, including age, sex, race, year of diagnosis, marital status at the time of diagnosis, origin recode NAACCR Hispanic Identification Algorithm (NHIA), primary site, T classification, N classification, TNM stage, surgery recode, chemotherapy recode, radiation recode, survival time, and survival outcomes were derived from the database. Age was categorized as ≤60 years, 6 -70 years, and >70 years.

### Study design

The propensity score matching (PSM) method was used to overcome patient selection bias. The following factors were matched between the two groups: age, sex, race, primary sites, T classification, N classification, TNM stage, marital status at the time of diagnosis, and origin recode NHIA. For each case in the surgery group, three cases in non-surgery group were randomly chosen for pairing by PSM. The non-SCLC cancer-specific survival outcomes were used to perform competing risk analyses.

Statistical analyses were performed using IBM SPSS Statistics 24.0 (IBM, Inc., Armonk, NY) and R version 3.5.1 (R Foundation for Statistical Computing, Vienna, Austria). Before and after matching, the characteristics between the two groups were compared using chi-square tests. A standardized difference less than 10% was acceptable to evaluate the balance of covariates before and after matching. The overall survival (OS) and the cause-specific survival (CSS) were estimated by the Kaplan-Meier method, and log-rank tests were used to analyze differences between curves. Univariate and multivariate Cox regression analyses were used to identify significant prognostic factors. Two-sided *P* values < 0.05 were considered statistically significant.

### Ethics statements

Ethics approval was exempted by the Ethics Committee of Zhongshan Hospital of Fudan University (Shanghai, China), as the SEER is a publicly available database, and data extracted from SEER were identified as nonhuman study.

## Results

### Patients characteristics

There were 85462 patients with SCLC in the SEER database between 2000 and 2015. After rigorous selection, 998 patients with stage IIB-IIIC SCLC who underwent surgery combined with chemotherapy or radiotherapy or both were included in the surgery group, and 17467 patients who only received chemotherapy or radiotherapy or both were included in the non-surgery group. After matching based on propensity scores, 2994 patients were selected and enrolled in the non-surgery group (Figure 1A). As shown in Table 1, compared to the patients who received non-surgical treatment, the patients who received surgery were more likely to be white and married, were more easily to develop tumors located in lower lobe, and had a higher tumor T classification, N classification and TNM stage (all *P* < 0.05). After matching, there were no significant differences between the two groups in terms of age, sex, race, origin record NHIA**,** marital status, primary sites, TNM stage, T classification, and N classification (all *P* > 0.05; Figure 1B).

### Survival analyses

**Figure 2:**
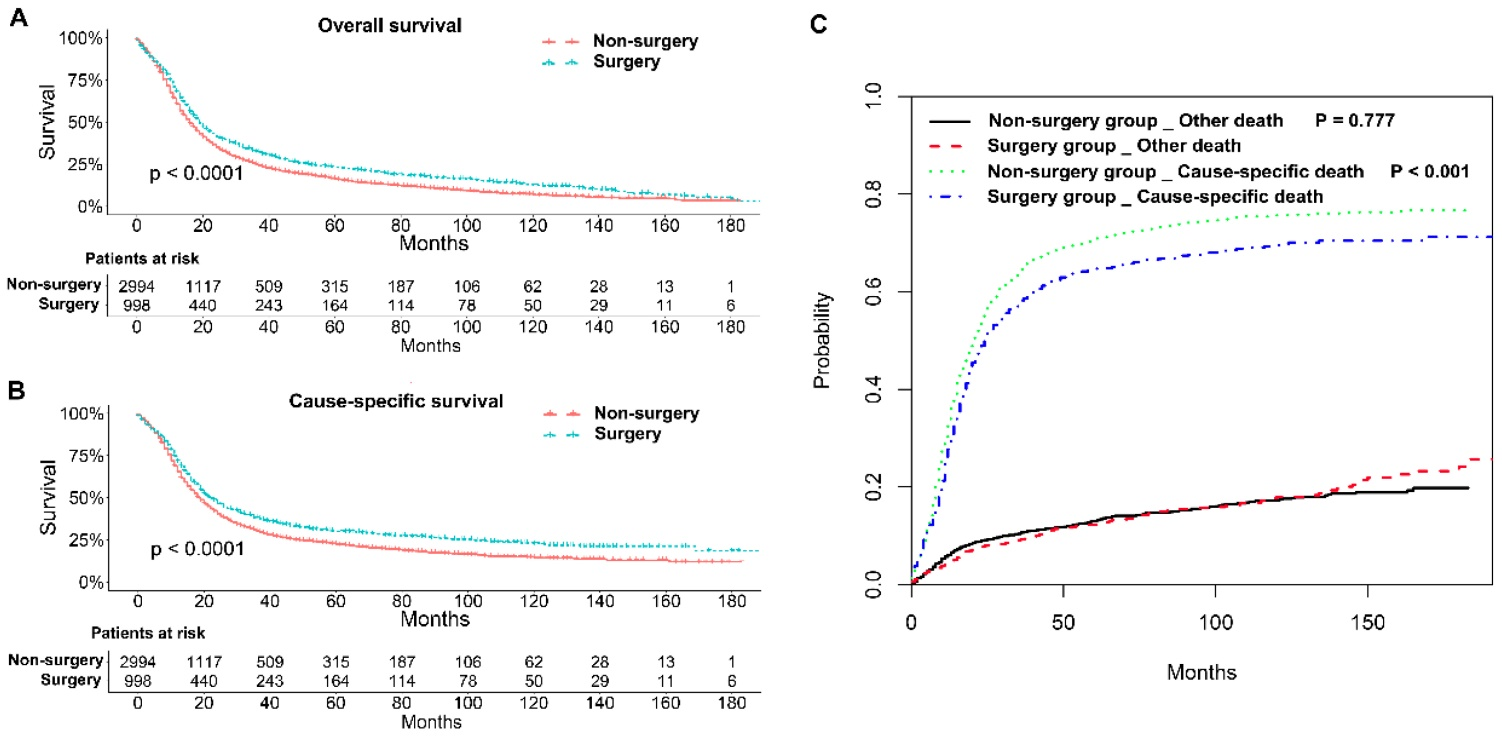
Survival analyses of patients with stage IIB-IIIC SCLC undergoing surgical treatment and non-surgical treatment. (A) Kaplan-Meier analysis of overall survival; (B) Kaplan-Meier analysis of cause-specific survival; (C) Competing risk analysis.

The 1, 2, and 3-year OS rates and CSS rates of patents with stage IIB-IIIC SCLC in the two groups were shown in Table 2. Generally, patients in the surgery group had better OS and CSS rates than those in the non-surgery group (*P* < 0.001; Figure 2A and B). Competing risk model analysis showed that the patients who received non-surgical treatment had a higher risk of cause-specific death from SCLC (*P* < 0.001), and there was no significant difference in the probabilities of other causes of death (*P* = 0.777; Figure 2C).

The correlation between survival and other parameters was further investigated. Univariate analyses showed that age (*P* < 0.001), sex (*P* < 0.001), marital status (*P* = 0.009), TNM stage (*P* < 0.001), T classification (*P* < 0.001), N classification (*P* < 0.001), and therapy methods (*P* < 0.001) were statistically significant predictors of OS (Table 3). There was no significance in terms of race (*P* = 0.131), origin record NHIA (*P* = 0.941), and tumor primary site (*P* = 0.763). Based on multivariate analyses, age (*P* < 0.001), sex (*P* < 0.001), T classification (*P* < 0.001), N classification (*P* = 0.004), and therapy methods (*P* < 0.001) were independent prognostic predictors for stage IIB-IIIC SCLC.

**Figure 3:**
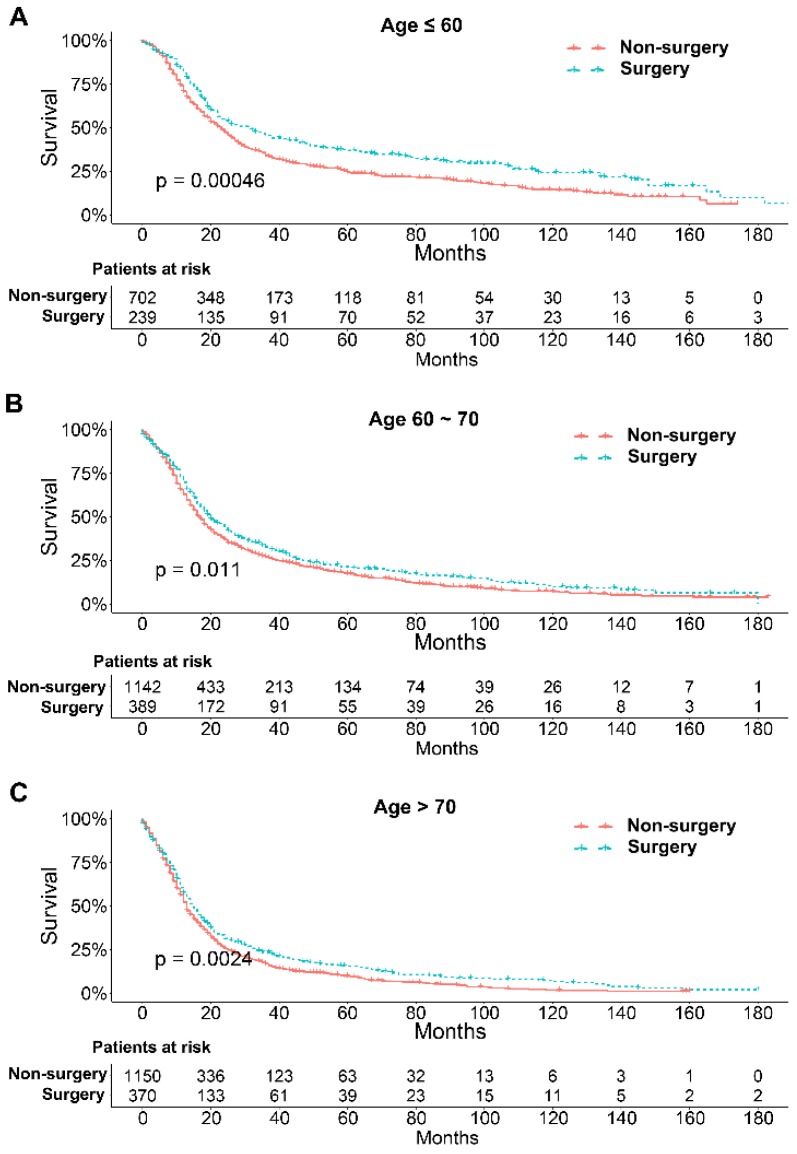
Survival analyses of patients undergoing surgical treatment and non-surgical treatment stratified by age after matching. (A) ≤ 60 years of age; (B) 60-70 years of age; (C) > 70 years of age.

**Figure 4:**
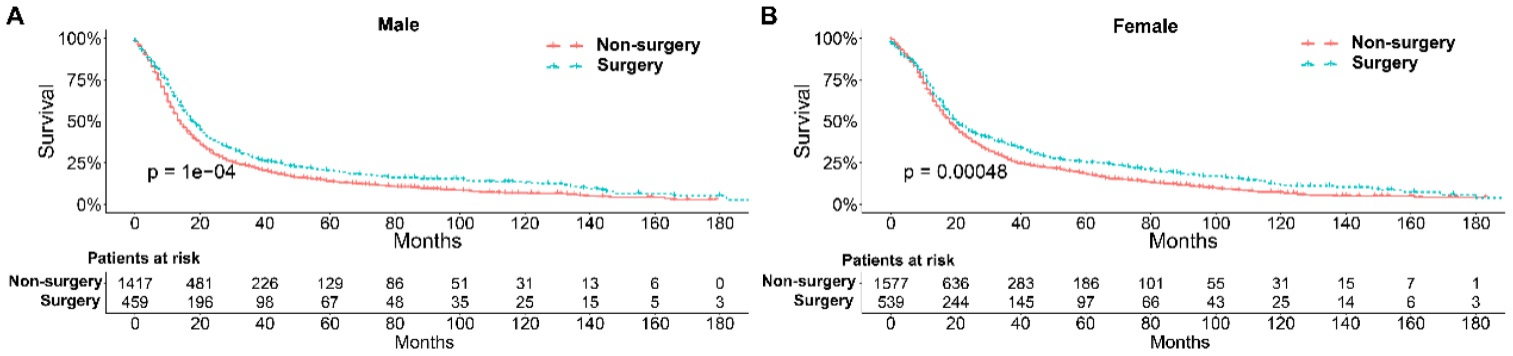
Survival analyses of patients undergoing surgical treatment and non-surgical treatment stratified by sex after matching. (A) male patients; (B) female patients.

**Figure 5:**
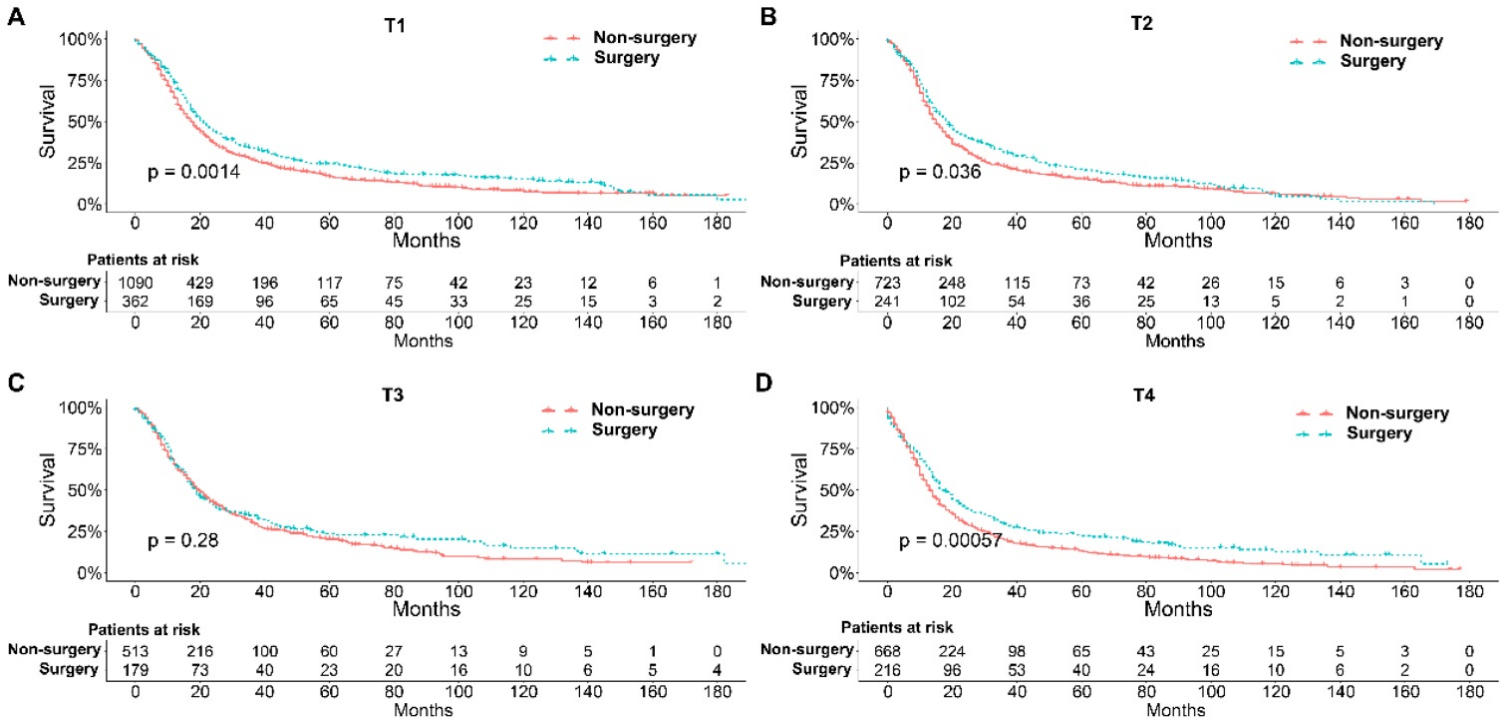
Survival analyses of patients undergoing surgical treatment and non-surgical treatment stratified by tumor T classification after matching. (A) T1 classification; (B) T2 classification; (C) T3 classification; (D) T4 classification.

**Figure 6:**
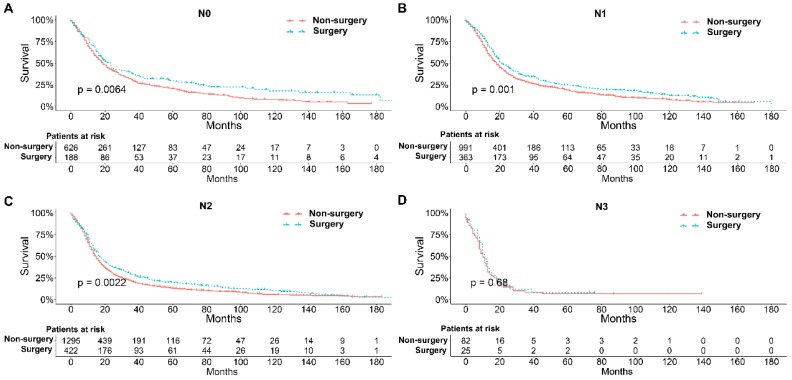
Survival analyses of patients undergoing surgical treatment and non-surgical treatment stratified by tumor N classification after matching. (A) N0 classification; (B) N1 classification; (C) N2 classification; (D) N3 classification.

**Figure 7:**
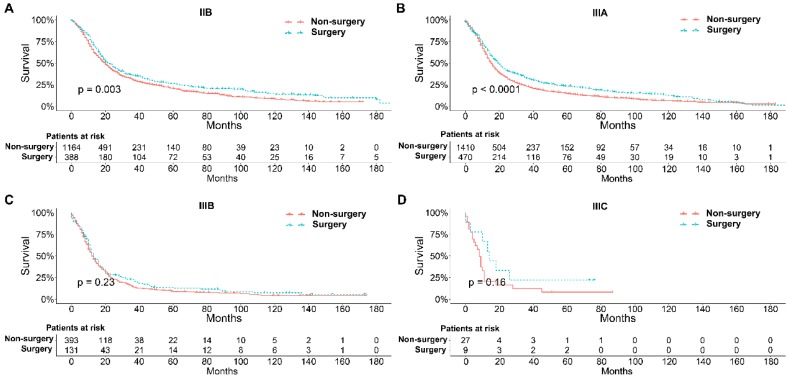
Survival analyses of patients undergoing surgical treatment and non-surgical treatment stratified by TNM stage after matching. (A) Stage IIB; (B) Stage IIIA; (C) Stage IIIB; (D) Stage IIIC.

To further explore the influence of therapy methods on the survival of stage IIB-IIIC SCLC patients, we stratified the matched patients by significant variables based on multivariate regression model analyses. In patients aged ≤ 60, 60-70, and >70 years, the survival rates were significantly better in the surgery group than in the non-surgery group (all P < 0.05; Figure 3A-C). Moreover, both male and female patients in the surgery group had better survival than those in the non-surgery group (all *P* < 0.001; Figure 4A and B). In the analysis of tumor T classification, only patients with T3 SCLC had comparable survival outcomes between the surgery group and the non-surgery group (*P* = 0.28), whereas there were significant differences in T1 (*P* = 0.0014), T2 (*P* = 0.036), and T4 (*P* < 0.001) SCLC (Figure 5A-D). With respect to the N classification, surgical treatment was associated with better survival than non-surgical treatment in N0 (*P* = 0.0064), N1 (*P* = 0.001), and N2 (*P* = 0.0022) SCLC, but not in N3 patients (*P* = 0.68; Figure 6A-D). For tumor TNM stage, a survival advantage related to surgery was observed in patients with stage IIB (*P* = 0.003) and IIIA (*P* < 0.001) SCLC (Figure 7A and B). However, no significant difference in survival was observed in patients with stage IIIB (*P* = 0.23) and IIIC (*P* = 0.16) SCLC (Figure 7C and D).

## Discussion

Previously, a large of studies including prospective and retrospective analyses focused on the treatment for NSCLC 8-15, because NSCLC accounted for 85% of lung cancer cases 16. It has been reported that surgery combined with chemotherapy/radiotherapy may improve the survival of the patients with advanced NSCLC 9, 15. For SCLC, the standard of care is chemotherapy and radiotherapy, even for limited disease 17. The role of surgery in the treatment of advanced SCLC is controversy 18-20. Several studies examined the role of surgery in the SCLC patients 21-23, but few studies solved the problem of potential selection biases in the analyses. In the present study, we used the PSM method to eliminate the selection bias by matching factors between the two groups, including age, sex, race, primary sites, T classification, N classification, TNM stage, marital status at the time of diagnosis, and origin record NIHA, and investigated the role of surgery in the chemotherapy/radiotherapy-based multimodality treatment of patients with stage IIB-IIIC SCLC. To our knowledge, it is the first time to use PSM method to eliminate the patient selection bias among the baseline variables of the two groups to investigate the role of surgery in the treatment for advanced SCLC. In addition, we used the competing risk model to distinguish cancer-specific death from death from other causes. No significant difference was found in the probabilities of other causes of death. These processes resulted in a more accurate comparison of survival outcomes between the two groups.

SCLC is a disease with a grave prognosis. In the present study, the 1, 2, and 3-year OS rates of patients with stage IIB-IIIC SCLC were 62.4%, 36.7%%, and 26.7%, respectively, which was consistent with the results of the study by Eberhardt et al. 24. Although surgery offers lung cancer patients the best chance of long-term survival, surgery alone is not sufficient, even for early stage SCLC. Analysis of the National Cancer DataBase indicated that patients with early SCLC who received surgery alone had a lower survival than those who received surgery combined with chemotherapy 25. A retrospective review by Szczesny et al. showed that surgery alone was associated with lower 5-year survival rates than a multidisciplinary approach for very limited disease 20. Furthermore, reports show that chemotherapy/radiotherapy may be effective and could decrease the risk of recurrence in SCLC, and could provide a survival benefit to patients 26, 27. However, chemotherapy/radiotherapy alone is associated with poor prognosis when compared with surgery with adjuvant therapy for early stage SCLC 28, 29. These results support an increased role of surgery in the multimodality treatment of SCLC, and the combination of surgery with chemotherapy/radiotherapy could be beneficial in patients with early stage SCLC. Increasing evidence indicates that multimodality treatment including surgical resection is associated with better survival than chemotherapy/radiotherapy alone 18, 30, 31. The treatment, namely surgery combined with adjuvant chemotherapy/radiotherapy, has a 5-year survival of approximately 40% -70% for limited-stage SCLC patients 26, 32.

In the past, surgery was only recommended for early stage SCLC 18, as it has little beneficial effects in patients with advanced disease. However, studies favor surgical intervention in patients with stage III SCLC. Shepherd et al. 33 showed that stage II (T1N1, T2N1) and stage III (any T3 or T1-2N2) SCLC patients who underwent surgery and adjuvant chemotherapy had a median survival of 72 weeks and 65 weeks, and projected 5-year survival rates of 24.5% and 24%, respectively. Eberhardt et al. 34 showed that the addition of surgery to multimodality treatment provided significant long-term survival, even in locally advanced patients with stage IIB/IIIA SCLC. Tsuchiya et al. 32 and Combs et al. 23 also reported comparable results. In the present study, OS and CSS were better in patients with stage IIB-IIIC SCLC in the surgery group than in those in non-surgery group, indicating that the inclusion of surgery in the multimodality treatment is beneficial for longer survival**.**

Survival differences stratified by significant variables were analyzed to investigate the impact of therapeutic approaches on the survival of matched patients receiving surgical treatment and non-surgical treatment. The results of multivariate Cox proportional hazards analyses indicated that age, sex, T classification, N classification, and therapy methods were significant independent predictors of survival outcome**.** Generally, elderly patients with SCLC had worse survival probably because they have a significantly higher number of comorbidities, smaller bone marrow reserve, and poor performance status that may affect the tolerance of chemotherapy 35, 36. Our results indicated that surgical treatment could be beneficial for patients with stage IIB-IIIC SCLC of all ages compared with non-surgical treatment. With respect to sex, men had higher smoking rates than women at any time point, which may contribute to the higher mortality rate 37. We showed that surgical treatment was associated with significantly better survival than non-surgical treatment for both male and female patients. Matched Kaplan-Meier survival analyses showed that surgery was associated with longer survival for T and N classification SCLC, although the difference was not significant for T3 (*P* = 0.28) and N3 (*P* = 0.68) classifications. The results were consistent with those of a study performed by Wakeam et al. 38. They showed that the survival differences favored surgery in any T classification and both N1 and N2 positive cohorts compared with non-surgical treatment, which indicated that surgery plays an important role in the multimodality treatment of SCLC. In our study, we also did a stratified analysis of survival by TNM stage between the two groups. Consistent with a previous study 38, we showed that surgery for stage IIB and IIIA SCLC was associated with improved long-term survival compared with nonsurgical treatment. Moreover, compared with previous studies, we also showed that surgery in the multimodality treatment had no influence on the survival of patients with stage IIIB and IIIC SCLC.

## Limitations

The present study had several limitations. First, the information available in the SEER database regarding chemotherapy and radiotherapy for SCLC was limited, and we were unable to obtain specific information regarding chemotherapy drugs, radiotherapy dose, and the treatment course. Second, the tumor differentiation in most cases in the non-surgery group was unknown, and the impact of tumor differentiation on survival outcomes could not be analyzed. Third, certain patients were excluded because of incomplete or inaccurate clinical information, which may lead to potential selection bias.

## Conclusions

The present study demonstrated that the combination of surgery with chemotherapy/radiotherapy was associated with better survival outcomes than conventional chemotherapy/radiotherapy alone in stage IIB and IIIA SCLC, but not in stage IIIB and IIIC SCLC. However, surgical resection was not associated with improved survival outcomes in the management of T3 classification and N3 classification.

## Figures and Tables

**Table 1:** Patient characteristics before and after matching

Characteristics	Before matching			After matching		
	Non-surgery	Surgery	*P*	Non-surgery	Surgery	*P*
Age (mean)	66.26	66.74	0.131	67.22	66.74	0.162
Age group			0.019			0.753
≤ 60	4896	239		702	239	
60-70	6363	389		1142	389	
> 70	6208	370		1150	370	
Sex			0.441			0.464
Male	8252	459		1417	459	
Female	9215	539		1577	539	
Race			0.001			0.757
White	15075	902		2723	902	
Black	1649	69		202	69	
Others	743	27		69	27	
NHIA			0.08			0.165
SHL	650	48		114	48	
NSHL	16817	950		2880	950	
Tumor Site			< 0.001			0.866
Upper lobe	9928	572		1726	572	
Middle lobe	829	55		141	55	
Lower lobe	3786	308		942	308	
Overlapping	281	22		70	22	
Lung, NOS	2643	41		115	41	
Marital status			0.001			0.815
Married	9014	572		1710	572	
Ever-married	5820	306		942	306	
Never-married	1985	81		243	81	
Unknown	648	39		99	39	
TNM stage			< 0.001			1.000
IIB	1284	388		1164	388	
IIIA	6667	470		1410	470	
IIIB	7747	131		393	131	
IIIC	1769	9		27	9	
T classification			< 0.001			0.934
T1	4189	362		1090	362	
T2	2282	241		723	241	
T3	2122	179		513	179	
T4	8874	216		668	216	
N classification			< 0.001			0.235
N0	1745	188		626	188	
N1	1600	363		991	363	
N2	11354	422		1295	422	
N3	2768	25		82	25	

NHIA: NAACCR Hispanic Identification Algorithm NSHL: Non-Spanish-Hispanic-Latino SHL: Spanish-Hispanic-Latino

**Table 2:** The survival rate in non-surgery group and surgery group

Survival rate	Total	Non-surgery group	Surgery group
1-year OS	62.4%	60.6%	67.8%
1-year CSS	66.9%	65.4%	71.5%
2-year OS	36.7%	35.1%	41.5%
2-year CSS	42.1%	40.4%	46.8%
3-year OS	26.7%	24.8%	32.6%
3-year CSS	32.2%	30.1%	38.0%

OS: overall survival CSS: cause-specific survival

**Table 3:** Univariate and multivariate analyses of overall survival of all patients after matching

Variables	Univariate Analysis			Multivariate analysis		
	HR	95% CI	*P*	HR	95% CI	*P*
Age			< 0.001			< 0.001
≤ 60	Reference	Reference		Reference	Reference	
60 - 70	1.370	1.247-1.506	< 0.001	1.369	1.245-1.506	< 0.001
> 70	1.850	1.684-2.033	< 0.001	1.865	1.694-2.053	< 0.001
Sex						
Male	Reference	Reference		Reference	Reference	
Female	0.835	0.779-0.896	< 0.001	0.818	0.760-0.880	< 0.001
Race			0.131			
White	Reference	Reference				
Black	0.896	0.778-1.031	0.126			
Others	0.849	0.672-1.072	0.169			
NHIA						
SHL	Reference	Reference				
NSHL	0.933	0.831-1.187	0.941			
Tumor Site			0.763			
Upper lobe	Reference	Reference				
Middle lobe	1.017	0.866-1.195	0.835			
Lower lobe	1.044	0.962-1.132	0.302			
Overlapping	1.107	0.829-1.479	0.491			
Lung, NOS	0.975	0.859-1.106	0.689			
Marital status			0.009			0.115
Married	Reference	Reference		Reference	Reference	
Ever-married	1.107	1.025-1.196	0.010	1.105	1.019-1.200	0.016
Never-married	0.894	0.780-1.024	0.105	1.028	0.896-1.179	0.698
Unknown	0.985	0.808-1.202	0.885	1.079	0.884-1.317	0.454
TNM stage			< 0.001			
IIB	Reference	Reference				
IIIA	1.206	1.117-1.302	< 0.001			
IIIB	1.540	1.380-1.717	< 0.001			
IIIC	1.898	1.321-2.728	< 0.001			
T classification			< 0.001			<0.001
T1	Reference	Reference		Reference	Reference	
T2	1.135	1.036-1.244	0.007	1.112	1.015-1.219	0.023
T3	0.946	0.851-1.051	0.299	1.134	0.986-1.291	0.058
T4	1.227	1.119-1.346	< 0.001	1.360	1.226-1.509	< 0.001
N classification			< 0.001			< 0.001
N0	Reference	Reference		Reference	Reference	
N1	1.027	0.930-1.135	0.596	1.207	1.064-1.370	0.004
N2	1.245	1.133-1.369	< 0.001	1.461	1.295-1.647	< 0.001
N3	1.966	1.582-2.444	< 0.001	2.134	1.697-2.685	<0.001
Therapy						
Non-surgery	Reference	Reference		Reference	Reference	
Surgery	0.809	0.746-0.878	< 0.001	0.807	0.744-0.876	< 0.001

## Abbreviations

SCLC: small cell lung cancer
SEER: Surveillance, Epidemiology, and End Results
NHIA: NAACCR Hispanic Identification Algorithm
NSHL: Non-Spanish-Hispanic-Latino
SHL: Spanish-Hispanic-Latino
PSM: propensity score matching
OS: overall survival
CSS: cause-specific survival
